# Bone impairment in atypical hemolytic and uremic syndrome treated by long-term eculizumab

**DOI:** 10.1007/s00467-024-06564-6

**Published:** 2024-10-18

**Authors:** Maitena Regnier, Anne-Laure Sellier Leclerc, Julie Tenenbaum, Marine Desjonqueres, Pascale Chavassieux, Véronique Fremeaux-Bacchi, Delphine Farlay, Justine Bacchetta

**Affiliations:** 1 Centre de Référence Des Maladies Rénales Rares, Centre de Référence Des Maladies Rares du Calcium Et du Phosphore, Filières Maladies Rares ORKID Et OSCAR, Hospices Civils de Lyon & Université Claude-Bernard Lyon 1, Lyon, France; 2 Service de Néphrologie, Rhumatologie Et Dermatologie Pédiatriques, Hôpital Femme Mère Enfant, Hospices Civils de Lyon, Boulevard Pinel, 69677 Bron Cedex, France; 3 Service de Néphrologie Pédiatrique, CHU de Montpellier, Montpellier, France; 4 INSERM, UMR 1033, Univ Lyon, Université Claude Bernard Lyon 1, 69008 Lyon, France; 5 Assistance Publique-Hôpitaux de Paris, Department of Immunology Biology, European Hospital Georges Pompidou, Paris, France; 6 INSERM, UMRS1138, Centre de Recherche Des Cordeliers, Team “Inflammation, Complement and Cancer”, Paris, France

**Keywords:** AHUS, Eculizumab, Bone, C3 deposits

## Abstract

Atypical hemolytic uremic syndrome (aHUS) is a thrombotic microangiopathy, related to complement dysregulation, including Factor H deficiency (FH) treated by lifelong eculizumab therapy. Its long-term tolerance is not yet fully described. We report two patients with genetic FH deficiency receiving long-term eculizumab and displaying a peculiar bone phenotype. First case is a 13-year-old girl, presenting with bone pains, arthritis, and deformities, for which X-rays and MRI described multifocal osteochondritis. Bone biopsy revealed an active remodeling bone (many areas of bone formation and resorption) and C3c accumulation on immunohistochemical staining. The second patient is an 11-year-old girl, displaying mechanical bone pains, for which bone scintigraphy found hypofixation of wrists and ankles. These findings could be consistent with a side effect of eculizumab, as C3c accumulation may result from the downstream C5-blockade. Alternatively, bone alterations could be due to the absence of FH, as described in murine models. Further investigations are required to characterize bone disease in aHUS.

## Introduction

Atypical hemolytic uremic syndrome (aHUS) is a rare thrombotic microangiopathy (TMA) caused by acquired or inherited complement dysregulation [1]. In pediatrics, etiologies are mainly genetic, factor H (FH) deficiency accounting for 20–30% of cases. FH deficiency is the most severe form of aHUS, associated with a high risk of relapses, chronic kidney disease (CKD) and kidney failure, and ultimately mortality, at least in the absence of targeted therapy [1]. Its management consists of chronic therapy with eculizumab, an anti-C5 monoclonal antibody, and more precisely a terminal complement-blocking antibody limiting the activation of the complement system. Eculizumab was approved in Europe in 2009 for aHUS and has strongly contributed to improve patients’ outcomes. It is well tolerated [2], but due to the rarity of the disease and the relatively recent approval, its long-term effects, if any, remain to be described. Here, we report on two teenagers with FH deficiency on long-term eculizumab therapy who present with a peculiar bone impairment.

## Case presentation

**Fig. 1:**
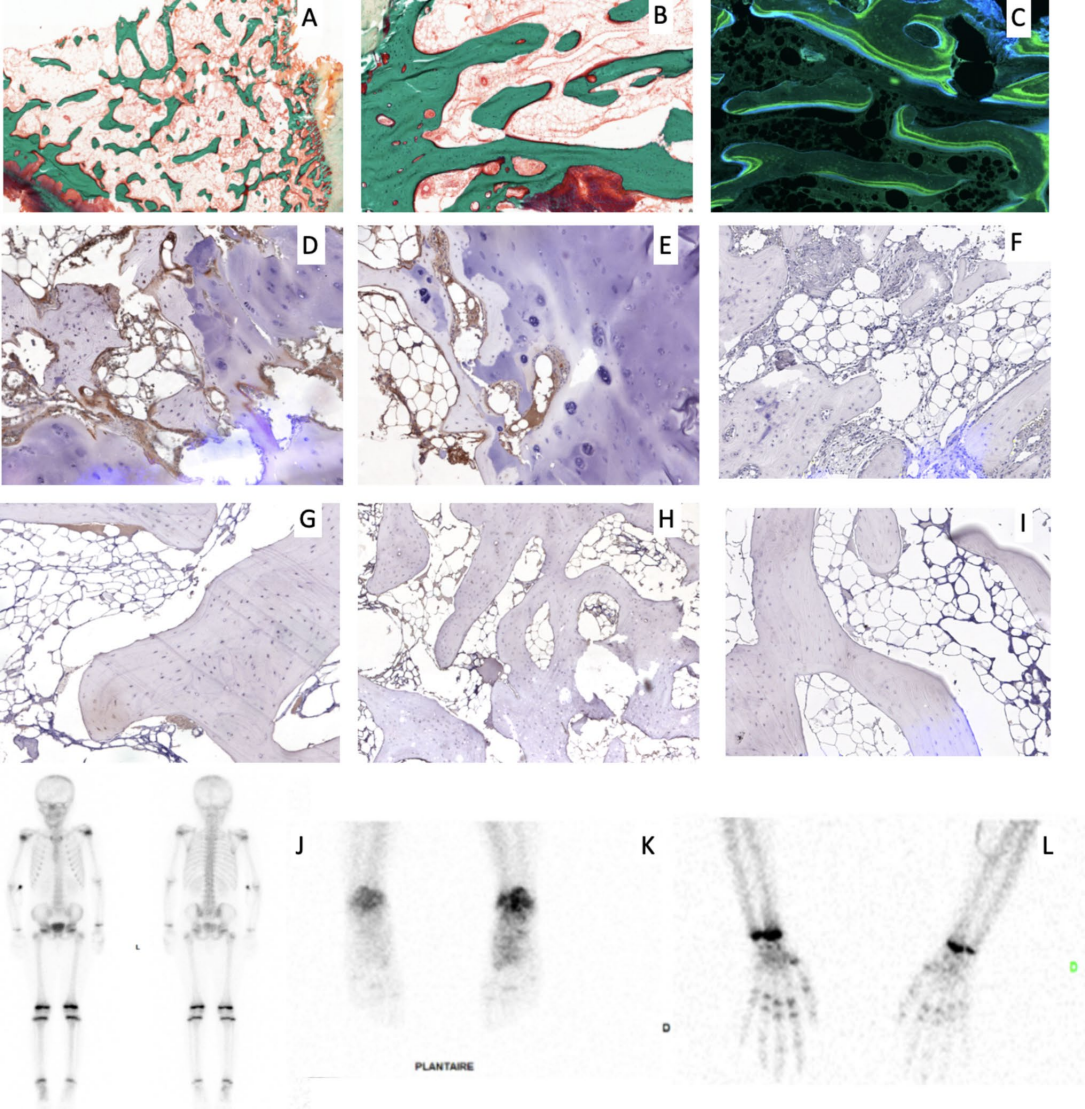
Bone abnormalities of the two patients. **A**–**C** Histological sections of healthy radius and pathologic ulna from patient 1. **A** Goldner-stained section showing the abundance of osteoid surfaces and adiposity of bone marrow of healthy radius, magnification × 2.5. **B** Goldner-stained section of pathological ulna. **C** Unstained section of healthy radius illustrating the extended labeled surfaces by tetracycline labels. **D**–**I** Immunostaining on PFA 4% fixed and paraffin-embedded bone sections with primary anti-C3c (**D**, **E**, **G**, **H**) or with control isotype antibody (**F**, **I**). Briefly, sections were deparaffinized and rehydrated, primary polyclonal rabbit anti-human C3c 1/2000 (ref 0062, Dako Glostrup Denmark) was incubated overnight and then incubated with secondary antibody HRP-conjugated goat anti-rabbit (Envision system, Dako Glostrup Denmark) for 1 h. After washing, sections were revealed by 3,3′-diaminobenzidine and counterstained with Mayer’s hematoxylin. **D** Healthy radius from patient 1, anti-C3c. **E** Pathological ulna from patient 1, anti-C3c. **F** Healthy radius from patient 1, isotype control. **G** Tibia fragment from control girl (10-year-old), anti-C3c. **H** Tibia fragment from control girl (14-year-old), anti-C3c. **I** Tibia fragment from control girl (10-year-old), negative control. In bone from patient 1 a strong staining for C3c was found in bone marrow and on bone surfaces, both in healthy radius and pathological radius (**D**, **E**). In contrast, only a weak staining for C3c was found in tibia from the 2 controls (**G**, **H**). No staining was found isotype control antibody (**F**, **I**). Magnification × s10 for **B**–**I**. **J**–**K** Bone scintigraphy of the second patient (**J**). Whole body scintigraphy. **K** Ankles scintigraphy. **L** Wrist scintigraphy. Bone scintigraphy described asymmetrical bone perfusion and metabolism, diffusely affecting the left foot and right hand

The first patient is a 13-year-old girl, presenting inherited FH deficiency aHUS since the age of 2 months, with arterial hypertension and stage 2 CKD (estimated glomerular filtration rate (eGFR) 78 mL/min/1.73 m^2^). She received eculizumab since 18 months of age (current dose 900 mg/2 weeks), with several unsuccessful withdrawal attempts. Around puberty, she displayed bone and joint pains, with arthritis, stiffness and deformities of ankles, knees, wrists, and elbows. X-rays and magnetic resonance imaging (MRI) described multifocal osteochondritis of unknown etiology. This patient received bisphosphonates and underwent ankles and elbow surgery, for severe substance loss and radial head dystrophy. Samples from the radius (healthy area) and ulna (pathological area) were performed during the surgery. Histological sections are presented in Fig. 1. There were neither TMA histological signs, and CKD-associated mineral and bone lesions were unlikely given the eGFR. In both endosteal and cancellous bone, increased bone formation was observed, as reflected by numerous active osteoblasts, high mineralization rate, and extensive osteoid surface (i.e., non-mineralized areas). There were also many areas of bone resorption, with numerous bone lacunae. Bone marrow was very adipose for age. These findings are consistent with an increased bone turnover without mineralization defect. An immunostaining study of complement was carried out, showing a significant accumulation of C3c. The choice of this antibody was made on the basis of complement study protocols commonly used in routine practice [3]. Because of this atypical presentation, a genome analysis was performed, which was normal except for the known heterozygous pathogenic variant in *CFH* gene (c.3572C > G p.Ser1191Trp), causing a functional rather than quantitative deficiency in FH. Symptoms stabilized at the end of pubertal spurt.

A chronic non-bacterial osteomyelitis could have been discussed, but a multi-disciplinary evaluation with experts from the French reference center for rare inflammatory diseases ruled out this diagnosis, based on the absence of both typical radiological and histological signs of the disease, notably the absence of inflammatory infiltrate on the biopsy.

The second patient is an 11-year-old girl, presenting aHUS due to complete inherited FH deficiency since the age of 11 days (homozygous c.3693_3696del ATAG p.X1232I fsX38), with an acute presentation associating acute anuric kidney failure, heart failure, and lactic acidosis. She was first treated by plasmapheresis; eculizumab was introduced at three weeks of age and then continued as maintenance therapy (current dose 900 mg/3 weeks). Around puberty, she also displayed non-inflammatory joint pains of ankles, knees, and wrists, with joint swelling, but neither deformity nor stiffness, with normal eGFR 107 mL/min/1.73 m^2^. Dual X-ray absorptiometry (DXA) revealed low bone mineral density both in the lumbar spine (Z-score − 2.6) and femoral neck (Z-score − 1.2). Bone scintigraphy, reported in Fig. 1, revealed asymmetrical bone perfusion and metabolism, diffusely affecting left foot and right hand. MRI was inconclusive, revealing no signal abnormalities in favor of osteitis or fracture, but describing a linear punctiform hypersignal in the calcaneus, consistent with residual islands of red marrow.

The two patients do not have exactly the same clinical presentation, and we did not have the opportunity to perform a bone biopsy in the second patient. However, she displays a peculiar bone phenotype; a multi-disciplinary evaluation did not find any other cause for this bone phenotype, which is intriguing and could be secondary to aHUS*.*

This retrospective case description was approved by the local IRB (*Comité d’Ethique des Hospices Civils de Lyon*, session 29/6/2022, 22_884). Figure 1 illustrates the most relevant radiological and pathological findings in these patients.

## Discussion

Here, for the first time, we report intriguing data on bone impairment in two teenagers with inherited FH deficiency receiving long-term maintenance eculizumab therapy. This peculiar bone phenotype associated pains, joint swelling, deformations, and stiffness. Initial investigations revealed osteochondritis with a highly remodeling bone and C3c accumulation on the biopsy for the first patient and hypofixation of two areas on the bone scintigraphy with low bone density on DXA for the second. Usual causes of osteochondritis were ruled out in the first patient, namely traumatic, genetic, mechanical, and metabolic causes; they were not consistent with the histological findings either. Osteochondrosis was initially suspected, as aHUS could have induced vascular bone lesions responsible for the bone phenotype, but bone biopsy revealed no vascular damage or evidence of TMA.

We also wondered whether the bone phenotype could be related to aHUS itself. This disease can indeed affect numerous organs, but bone disorders have not yet been described. That said, in addition to its immune functions, the complement system plays many other roles, notably in tissue regeneration and homeostasis, including in the skeletal system [4]. It is indeed implicated in bone growth, bone cell differentiation, and turnover [4], and specifically influences osteoblastic and osteoclastic activity. Its major role in bone maturation and growth could explain skeletal disorders in aHUS in which it is dysregulated, and more particularly at the time of pubertal bone formation peak, as seen here. More specifically, FH per se plays a specific role in bone architecture, as demonstrated in the FH^−/−^ murine models [5]. Indeed, tridimensional reconstruction of their femur revealed reduced cortical thickness and tissue mineral density, with a significant increase in marrow area and bone porosity compared to controls. The study of cultured osteoblasts and osteoclasts extracted from bone marrow FH^−/−^ mice showed increased numbers of osteoblasts and osteoclasts, as well as increased osteoclastic function. These abnormalities were observed at 16 weeks of age, before the onset of CKD. All these histological features may correspond to those observed in the patient’s biopsy, which could therefore be secondary to the absence of FH. However, this would not explain C3 accumulation that we observed both on healthy and pathological areas (as compared to bone from an age-matched healthy control from the INSERM lab biocollection).

Thus, we wondered whether our results could also be consistent with a side effect of eculizumab therapy, as the C3c accumulation may result from the downstream C5 blockade. With this hypothesis, it remains to be demonstrated whether C3c accumulation in bone can be deleterious per se. In vitro cell cultures of human osteoblastic and osteoclastic cells revealed that osteoclastogenesis was significantly induced by C3a and C5a, whereas osteogenic differentiation was not affected [6]; of note, C3c was unfortunately not tested in this study, to identify potential direct effects of this component on bone dynamics. It should be noted that two opposing effects on osteogenesis secondary to eculizumab treatment can be identified here: eculizumab may increase C3a and decrease C5a due to inhibition of C5 cleavage into C5a and C5b. Yet, both C3a and C5a have been described as promising for osteoclastogenesis, as indicated above. However, in our bone sections, we observed rather increased osteoclastogenesis. Our results are too preliminary to know whether this balance in favor of osteoclastogenesis is secondary to eculizumab, inherent in the FH deficiency itself, or whether the effect of the increase in C3a is predominant over the decrease in C5a on osteoclasts.

Bony side effects of eculizumab therapy have not been described, but we may not yet have the necessary hindsight to objectify possible long-term side effects of the drug. The evaluation of safety and efficacy of eculizumab by the international working group has nevertheless identified a wrist fracture in one patient [2], but this was classified as unrelated. The data presented here are still too preliminary to formally conclude that eculizumab could induce bone lesions, but long-term monitoring seems necessary, especially since France was one of the first countries in the world to have routine access to eculizumab.

In conclusion, we report for the first time two cases of aHUS teenagers with inherited FH deficiency receiving long-term eculizumab and presenting peculiar bone lesions. These findings could be consistent with a side effect of the drug but also a consequence of FH deficiency itself. As this disease is quite rare, and this bone phenotype (linked either to the pathology itself or to its treatment) could be even rarer, we have asked our French colleagues through the French Society of Pediatric Nephrology: no other cases were identified; as such, in the future, a European survey on the topic will be of interest to identify other cases and better understand the underlying pathophysiology of this peculiar skeletal impairment in aHUS.

## Summary

### What is new?

- We report cases of FH deficiency receiving eculizumab and presenting a previously undescribed bone phenotype. These findings could be secondary to a side effect of the drug, but also a consequence of FH deficiency itself.

## Data Availability

The data that support the findings of this study are available on request from the corresponding author.

## Abbreviations

aHUS: Atypical hemolytic uremic syndrome
CKD: Chronic kidney failure
FH: Factor H
GFR: Glomerular filtration rate
MRI: Magnetic resonance imaging
TMA: Thrombotic microangiopathy
